# Native and Non-Native Egg Parasitoids Associated with Brown Marmorated Stink Bug (*Halyomorpha halys* [Stål, 1855]; Hemiptera: Pentatomidae) in Western Slovenia

**DOI:** 10.3390/insects12060505

**Published:** 2021-05-31

**Authors:** Mojca Rot, Lara Maistrello, Elena Costi, Iris Bernardinelli, Giorgio Malossini, Luca Benvenuto, Stanislav Trdan

**Affiliations:** 1 Institute of Agriculture and Forestry Nova Gorica, Pri hrastu 18, 5000 Nova Gorica, Slovenia; 2 Dipartimento di Scienze della Vita, Università di Modena e Reggio Emilia, 42122 Reggio Emilia, Italy; lara.maistrello@unimore.it (L.M.); elecosti@unimore.it (E.C.); 3 ERSA—Regional Agency for Rural Development—Plant Health Service, 33050 Pozzuolo del Friuli, Italy; iris.bernardinelli@ersa.fvg.it (I.B.); giorgio.malossini@ersa.fvg.it (G.M.); luca.benvenuto@ersa.fvg.it (L.B.); 4 Department of Agronomy, Biotechnical Faculty, University of Ljubljana, 1000 Ljubljana, Slovenia; Stane.Trdan@bf.uni-lj.si

**Keywords:** *Halyomorpha halys*, biological control, egg parasitoids, *Anastatus*, *Trissolcus*, Slovenia

## Abstract

**Simple Summary:**

*Halyomorpha halys*, the brown marmorated stink bug (BMSB), is an invasive pest causing serious damage to agricultural production. Managing this pest species is challenging because of its wide host range and lack of effective control measures. Biological control of *H. halys* through natural enemies seems to be the most environmentally friendly and sustainable solution. Extensive knowledge of the native egg parasitoid fauna is needed prior to the introduction of a biological control program. The main purpose of the study, carried out in the Goriška region of Western Slovenia, was to detect egg parasitoid species associated with *H. halys* and to evaluate their impact on the pest population under local environmental conditions. High species richness was identified during the study, and five egg-parasitoids were recorded for the first time in Slovenia. The native species *Anastatus bifasciatus* dominated in urban and suburban areas, while the non-native *Trissolcus mitsukurii* prevailed in agricultural areas. Rapid recruitment of native parasitoids, the presence of an effective alien parasitoid species in the region and increasing overall parasitism rates are very encouraging results and valuable information for future activities regarding the biological control of *H. halys* in Slovenia.

**Abstract:**

*Halyomorpha halys* (Hemiptera: Pentatomidae), native to East Asia, has become a globally invasive pest, as a serious threat to agricultural production and a notorious nuisance pest in urban areas. Considerable efforts have been made so far to develop effective pest control measures to prevent crop damage. Biological control of this invasive stink bug by egg parasitoids has proven to be the most environmentally sustainable long-term solution. Knowledge of the native egg parasitoid fauna is of key importance when implementing a biological control program. Therefore, the main objective of our study was to detect egg parasitoid species associated with *H. halys* in the Goriška region (Western Slovenia) and to evaluate their impact on the pest population under field conditions. In the years 2019 and 2020, around 4600 *H. halys* eggs were collected in the wild and more than 3400 sentinel eggs were exposed to detect parasitoids in the field. Five egg-parasitoid species emerged from *H. halys* eggs: *Anastatus bifasciatus* (Hymenoptera: Eupelmidae), *Telenomus* sp., *Trissolcus basalis*, *Trissolcus mitsukurii* (Hymenoptera: Scelionidae) and *Ooencyrtus telenomicida* (Hymenoptera: Encyrtidae), all of them are new records for Slovenia. The native species, *An. bifasciatus*, dominated in urban and suburban areas, while non-native *Tr. mitsukurii* prevailed in agricultural areas. Overall parasitism rates of naturally laid eggs by the parasitoid species complex in 2019 and 2020 was 3.0 and 14.4%, respectively. Rapid recruitment of native parasitoids, early detection of an effective alien parasitoid species and increasing overall parasitism rates are very encouraging results, which need to be followed and verified in future research.

## 1. Introduction

The brown marmorated stink bug (BMSB) (*Halyomorpha halys* (Stål); Hemiptera: Pentatomidae) is an East Asian species, native to Japan, Korea, China and Taiwan [1]. This polyphagous species, with more than 100 host plants reported [2], has become a worldwide invasive pest, causing severe damage in agricultural production [1,3,4,5,6,7]. In the mid-1990s, it was introduced into Pennsylvania (USA) [3], and within a few years, it rapidly spread across the United States and Canada [8]. The same scenario of invasion occurred after its introduction in Europe in 2004 [9]. In 15 years, *H. halys* managed to colonize almost all climatically suitable areas in Europe [10,11,12,13,14,15,16,17,18,19,20,21,22] and became one of the most serious agriculture pests, as well as a nuisance insect in urban environments [23]. Due to the damage caused by *H. halys* and subsequent economic losses, the use of insecticides has substantially increased in commercial orchards [24], disrupting established integrated pest management programs for different crops in the USA and Europe [25,26,27]. More frequent pesticide application, including broad-spectrum insecticides, has affected beneficial arthropods and caused secondary pest outbreaks [27]. *Halyomorpha halys* management has become a great concern and challenge for European farmers. Italy was the first European country to suffer great damage in agriculture production due to attacks of this invasive pest. In 2019 estimated losses in fruit production in Northern Italy were almost 600 million EUR [28].

Many research groups and technicians around the world have been working feverishly over the last 10 years to find effective, feasible and environmentally acceptable ways of managing this pest. Exclusion nets have proven to be a promising tool to prevent *H. halys* from damaging nectarines and apples in Italy. Although they are expensive to purchase and deploy, the use of such nets is considered to be a cost-effective control strategy [29]. The attract-and-kill strategy was effective at managing low to moderate *H. halys* populations in apple orchards in Mid-Atlantic states in the USA [26]. Long-lasting insecticide-treated nets (LLINs) were tested in pear orchards in Central Italy. Although the method was not proven for *H. halys* control itself, it is proposed as part of an IPM program, which contributes to a significant reduction of pest population in the crop [30].

In past years, much attention has been given to alternative methods for long-term management. Biological control of *H. halys* with egg parasitoids is considered one of the most viable long-term solutions [24]. In its native range, *H. halys* is primarily regulated by several egg parasitoids belonging to genera *Trissolcus* Ashmead, *Telenomus* Haliday (Hymenoptera: Scelionidae), *Ooencyrtus* Ashmead (Hymenoptera: Encyrtidae) and *Anastatus* Motschoulsky (Hymenoptera: Eupelmidae) [31,32]. *Trissolcus japonicus* (Ashmead) is the most predominant and effective parasitoid in China, with high parasitism rates ranging from 50% to 90%, while *Trissolcus mitsukurii* (Ashmead) is the main parasitoid in Japan [1,31,32]. Numerous studies on natural enemies have been carried out in the USA and Europe [33,34,35,36,37,38]. Overall findings show that three principal groups of hymenopteran parasitoids are able to attack *H. halys* eggs in invaded areas: Scelionidae (*Telenomus*, *Trissolcus*, and *Gryon* spp.), Eupelmidae (*Anastatus* spp.), and Encyrtidae (*Ooencyrtus* spp.). However, so far, the level of parasitism by native species is too low to reduce pest population below economic thresholds [34,39]. *Anastatus bifasciatus* (Geoffroy) is the most widespread native egg parasitoid in Europe capable of developing on viable *H. halys* eggs [33,36,39,40,41] and is considered as the most suitable candidate for augmentative biological control in Europe. The first findings of adventive populations of *Tr*. *japonicus* occurred in Switzerland in 2017 and in Northern Italy in 2018 [42,43], where another important *H. halys* natural enemy, *Tr. mitsukurii*, was found in Friuli Venezia Giulia, the northeastern Italian region bordering Slovenia, and have brought new prospects for biological control of *H. halys* in Europe [43,44].

Native natural enemies of stink bugs (Hemiptera: Pentatomidae) are poorly investigated in Slovenia. Among egg parasitoids, the only record is of *Trissolcus scutellaris* (Thomson) parasitizing eggs of *Eurydema ventralis* (Kolenati) [45]. *Halyomorpha halys* was first detected in Western Slovenia in 2017 [22], and since then, it has caused considerable damage to fruit production [46]. Therefore, the aim of this study was to verify the presence and impact of parasitoids and predators of the eggs of this invasive pest in the region. During the surveys, special attention was paid to habitat investigation in the border area with Italy due to the higher probability of detecting non-native parasitoids of the genus *Trissolcus* that were recorded there [43,44].

## 2. Material and Methods

### 2.1. Field Survey of Native and Non-Native Parasitoids of Halyomorpha halys

Field surveys of parasitoids were carried out in 2019 and 2020 in the Goriška region of Western Slovenia. The monitoring of naturally laid egg masses was performed weekly, from June to the end of September. Locations were selected based on the presence and abundance of *H. halys* and preferred host plant availability. Different types of locations were inspected: agricultural areas with intensive fruit orchards and row crops, including hedges and shrubs along roads, parks and garden vegetation within urban areas (Table 1). The undersides of leaves were inspected throughout the canopies of trees and shrubs at a height between 0.5 to 2.0 m from the ground. Leaves with naturally laid egg masses were removed and placed in plastic Petri dishes. Data on location, host plant and date of collection were recorded for each egg mass. Samples were marked and transferred to the laboratory. The detailed examination of egg masses was performed under a stereomicroscope. The number of intact eggs and the number of eggs with signs of damage by sucking and chewing predators were counted. Predation damage on eggs was identified and classified according to Morrison et al. [47]. Hatching took place in growth chambers at 25 ± 1 °C, 60% ± 10% r.h. and L16:D8 photoperiod. The emergence of nymphs and parasitoids was recorded every second day, and the emerged nymphs and parasitoids were counted and removed from the Petri dishes. The parasitoids were kept in 96% ethanol until identification. Unhatched eggs were dissected, and eggs containing partially developed parasitoids were classified as parasitized. If the cause of mortality could not be diagnosed, eggs were counted as unhatched. When parasitoids had already emerged in the field, to determine the parasitoid previously contained, shapes of exit holes and meconia were examined and compared to the descriptions of Sabbatini-Peverieri et al. [48,49].

### 2.2. Laboratory Rearing of Halyomorpha halys and the Field Exposure of Fresh Sentinel Eggs

Adult stink bugs were collected from wild-growing and cultivated host plants in gardens and orchards in the vicinity of Nova Gorica (Western Slovenia) in the first half of May 2019 and 2020. The colonies were kept in ventilated plastic boxes with a solid bottom and mesh windows on all sides. The rearing diet composed of green beans, sunflower seeds, peanuts and pieces of carrots and was changed twice a week. To maintain humidity and provide water, cotton balls soaked in water were placed in the boxes. A layer of white polypropylene row cover was fixed under the mesh lids as an oviposition substrate. Egg masses were collected daily. Field exposure of fresh sentinel eggs was performed in two different locations: (i) suburban areas with hedges and shrubs in proximity to orchards and vineyards; (ii) native hedging close to intensive fruit orchards. Freshly laid egg masses (less than 24 h) were stapled to the underside of the leaves of different wild-growing plants (*Cornus sanguinea* L., *Corylus avellana* L., *Parthenocissus quinquefolia* (L.) Planch., *Acer negundo* L., *Vitis vinifera* L.) and remained exposed for 34 days. In 2019, egg masses were exposed once a week in June and within the first half of July. In 2020 the egg masses were exposed twice a week from the end of May to the end of June and from the end of July to the end of August. Collection and laboratory examination of egg masses followed the same procedure as described in Section 2.1.

### 2.3. Parasitoid Identification

A Nikon SMZ-2B stereo microscope (Nikon Corporation, Tokyo, Japan) with magnification up to 100× was used for morphological diagnosis. For a detailed examination of the microsculpture, which is essential for the determination of the species level, a spotlight in combination with a Mylar shield was used. Morphological characteristics of antennal sensilla were investigated under a compound microscope Nikon Eclipse Ni-U (Nikon Corporation, Tokyo, Japan). *Anastatus bifasciatus* was identified according to Askew and Nieves-Aldrey [50] and Peng et al. [51], *Ooencyrtus telenomicida* (Vassiliev) according to Samra et al. [52] and Triapitsyn et al. [53]. For *Telenomus* and *Trissolcus* the following keys were used; Johnson [54,55], Talamas et al. [56,57], Balusu et al. [58], Tortorici et al. [59] and Sabbatini Peverieri et al. [43]. *Trissolcus mitsukurii* was also compared with samples of *Tr. mitsukurii* collected in Friuli Venezia Giulia and kindly provided by the Regional Agency for Rural Development (ERSA), Italy.

### 2.4. Evaluation of Parasitoid Efficiency

Parasitoid efficiency evaluation was performed according to Bin and Vinson [60]. The parasitoid impact or parasitism rate was calculated for each location and year as the number of parasitized eggs over the total number of field-collected eggs (Table 2). Discovery efficiency, which describes parasitoid ability to find egg masses, was calculated as the number of egg masses discovered by the parasitoid over the total number of egg masses. Parasitoid exploitation efficiency was calculated as the number of parasitized eggs over the total number of eggs within the parasitized egg masses. The same evaluation method was applied to sentinel egg masses (Table 3).

## 3. Results

### 3.1. Naturally Laid Egg Masses Collected in the Field

In 2019 and 2020, a total of 174 *H. halys* egg masses were found in 11 different locations (Table 2). In 2019, 19 egg masses were found in total at five different locations and from only one egg mass collected on *Vitis vinifera*, parasitoids successfully emerged. They were all identified as *An. bifasciatus*, which were recorded for the first time in Slovenia. In 2020, 155 egg masses were found at 11 different locations.

**Figure 1:**
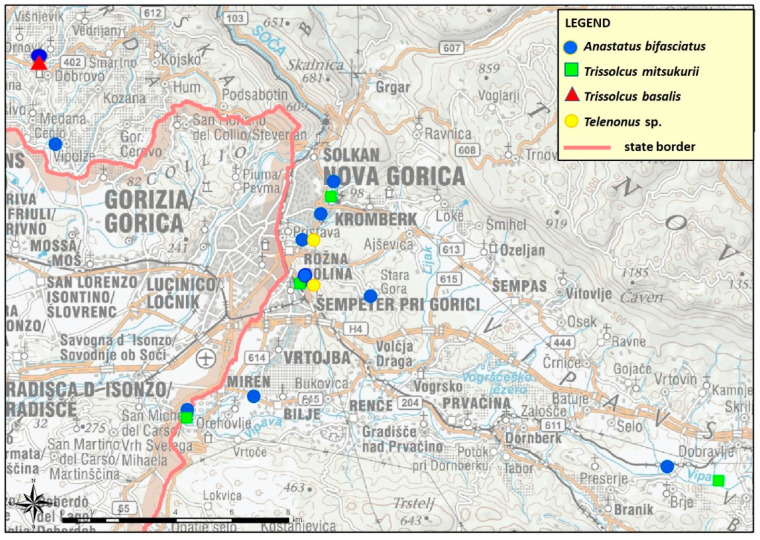
Map of *Anastatus bifasciatus*, *Trissolcus mitsukurii*, *Trisssolcus basalis* and *Telenomus* sp. records in Western Slovenia.

Among the 316 parasitoid wasps that emerged from the eggs, the most abundant species was *An. bifasciatus* (213 specimens), representing more than two-thirds of all parasitoids collected in the field. *Anastatus bifasciatus* was also the most widespread species since it was found at 9 out of 11 locations inspected in Westen Slovenia (Figure 1).

The second most abundant species was the non-native *Tr. mitsukurii* (77 specimens), collected at three different locations. The other two scelionid parasitoids occurred in smaller numbers: *Telenomus* sp. was found in two locations, and a single egg mass with *Trissolcus basalis* (Wollaston) was found at another location. All of these scelionids were identified for the first time in Slovenia.

**Figure 2:**
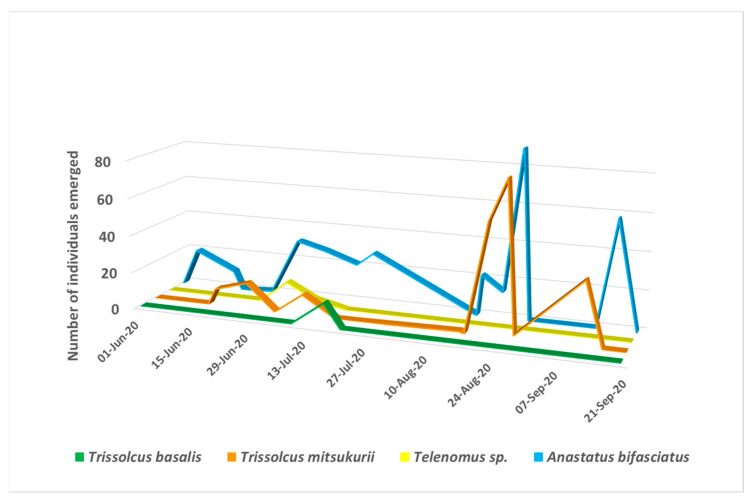
Seasonal occurrence of *H. halys* egg parasitoids observed in the Goriška region (Western Slovenia) in 2020.

The four egg parasitoids observed in 2020 showed a different seasonal occurrence (Figure 2). While *Telenomus* sp. and *Tr. basalis* were observed once in the season, from early to mid-July, *An. bifasciatus* was present throughout the whole season with two noticeable peaks, in the second half of July and in the second half of August. *Trissolcus mitsukurii* first appeared in mid to late June in small numbers, and the peak occurred in the second half of August.

**Figure 3:**
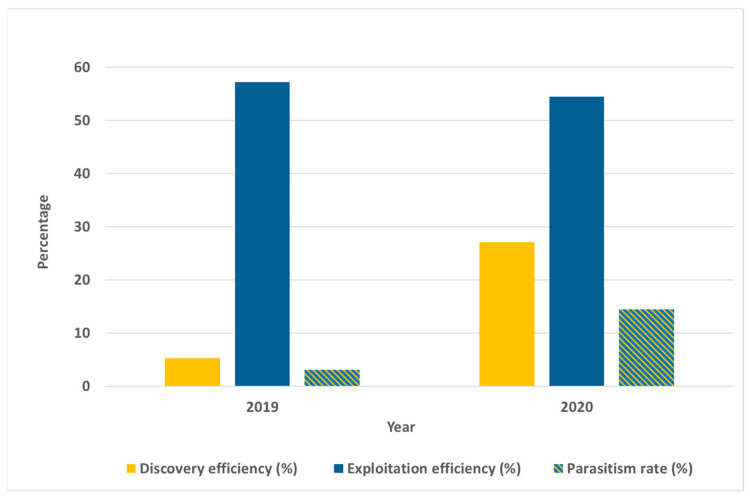
Parasitoid efficiency in parasitizing naturally laid *H. halys* egg masses in the Goriška region (Western Slovenia) in years 2019 and 2020.

In 2020, the overall parasitism rate was 14.4%, noticeably higher than in 2019 (3.0%). The increased impact of parasitoids in 2020 was also confirmed by higher discovery efficiency (27.1%) and a high level of exploitation efficiency (54.5%) (Figure 3). In 2019 and 2020, the overall predation rates were 6.2 and 5.6%, respectively (Table 2).

### 3.2. Sentinel Egg Masses Exposure

**Figure 4:**
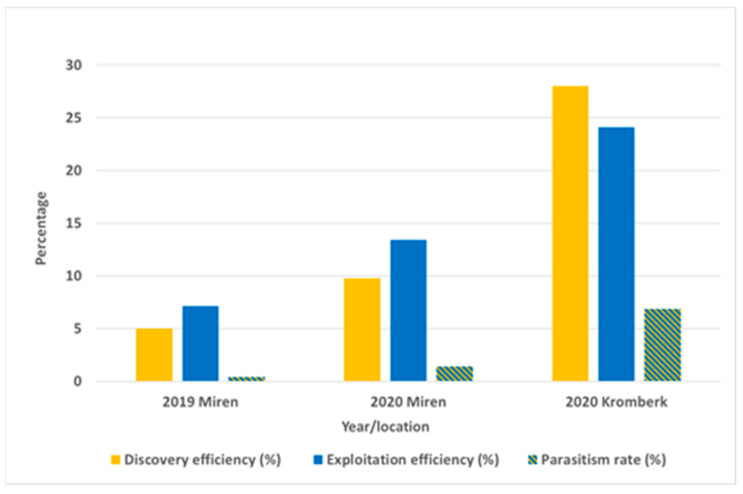
Parasitoid efficiency in parasitizing sentinel *Halyomorpha halys* egg masses exposed in the Goriška region (Western Slovenia) in the years 2019 and 2020.

In 2019 and 2020, 131 egg masses (3,456 eggs) were exposed at two different locations. In 2019 (40 egg masses; 1058 eggs), we exposed them in suburban and agricultural areas. The observed parasitoid impact was almost negligible in both locations (Table 3). No parasitoids emerged from the eggs exposed in the suburban area, whereas in the agricultural area, one egg mass was parasitized by *Oo. telenomicida*, which was detected for the first time in Slovenia. In 2020, 91 egg masses (2417 eggs) were exposed. A total of 107 adult parasitoids emerged from the eggs, with 65% of *Tr. mitsukurii* and 35% of *An. bifasciatus*. In the suburban area, the parasitism rate was 6.86%, with *Tr. mitsukurii* as the most abundant species, followed by *An. bifasciatus* (Table 3). The discovery and exploitation efficiencies were 28.0% and 24.1%, respectively. All three parasitism efficiency indices were higher compared with the agricultural area (Figure 4).

In the agricultural area, the total parasitism rate was 1.39%, and only *An. bifasciatus* was found (15 adults). In both years, predators demonstrated a greater impact on sentinel egg masses (ranging from 13.9% in 2019 to 15.7% in 2020) than parasitoids (Table 3).

### 3.3. Oviposition Hosts

**Figure 5:**
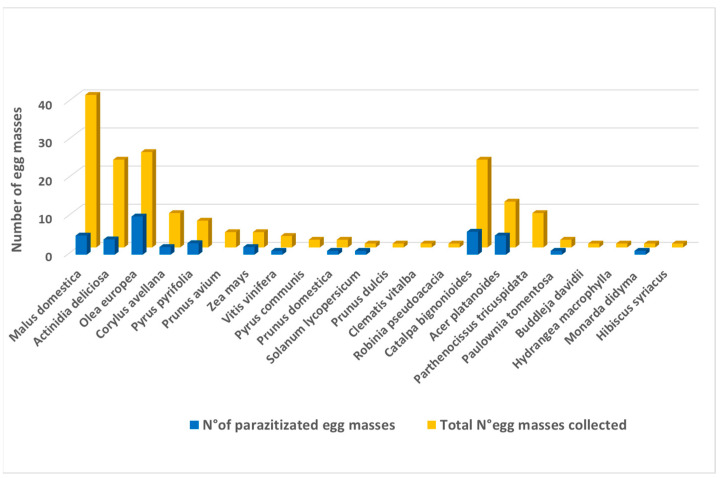
*Halyomorpha halys* oviposition host diversity and parasitoid efficacy by host.

During the survey, a total of 22 *H. halys* oviposition hosts were recorded (Figure 5). *Malus domestica* Borkh., *Actinidia deliciosa* (A.Chev.) C.F.Liang & A.R.Ferguson, *Olea europea* L., *Corylus avellana*, *Pyrus pyrifolia* (Burm.f.) Nakai and *Zea mays* L. were the most common oviposition hosts in the agricultural area. Among the fruit crops, apple was the main host, with 41 egg masses collected, almost a quarter of all *H. halys* egg masses collected in the region (Figure 5).

**Figure 6:**
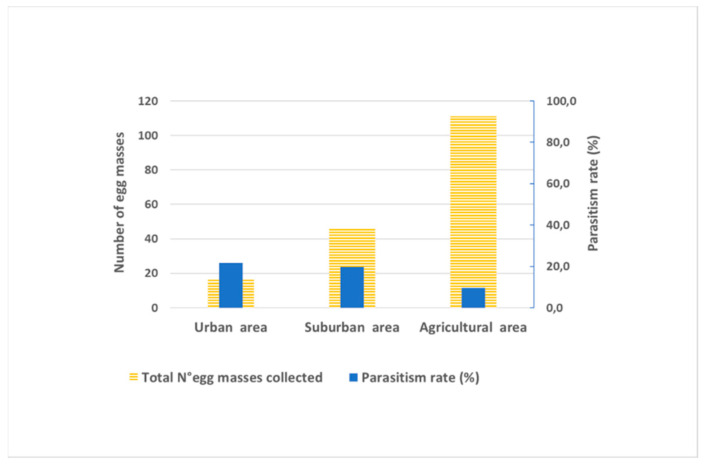
Parasitoid impact in different landscapes in Western Slovenia.

The parasitoid community associated with apple was composed of *An. bifasciatus* and *Tr. mitsukurii* in equal proportions, with an overall parasitism rate of 8.6%. The second was an olive tree with 24 egg masses collected and with a noticeably higher overall parasitism rate (15.3%) than found on the apple (Table S1. *Anastatus bifasciatus* was the predominant species and a major contributor to the total parasitism on olives, while *Tr. mitsukurii* had a minor part. The overall parasitism rate in agricultural areas was 9.7% (Figure 6).

**Figure 7:**
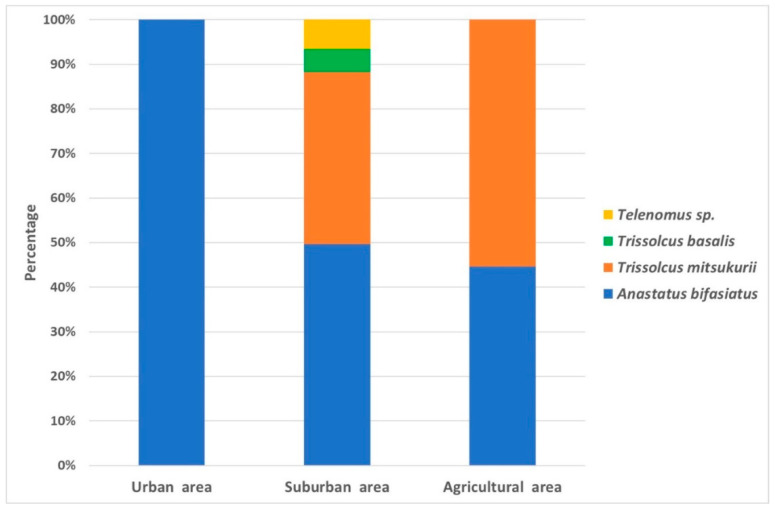
Parasitoid occurrence in different landscapes in Western Slovenia.

The most suitable oviposition hosts in the urban area were *Catalpa bignonioides* Walter and *Acer platanoides* L., where 20.8% of the eggs were parasitized by *An. bifasciatus* (Figure 7). Host plant diversity and parasitoid species richness were the highest in the suburban area, where *An. bifasciatus* and *Tr. mitsukurii* were the most abundant species, while *Telenomus* sp. and *Tr. basalis* together represented approximately one-tenth of the total population. Most of the parasitized eggs were found on *O. europea*, *A. deliciosa*, *P. domestica* and *P. tomentosa*. *Parthenocissus tricuspidata* (Siebold & Zucc.) Planch. is a good *H. halys* oviposition host but is apparently not attractive to parasitoids.

## 4. Discussion and Conclusions

In a two-year field survey conducted in 2019 and 2020 in the Goriška region (Western Slovenia), we identified the presence of different egg parasitoids of the invasive *H. halys*, including one non-European species. *Halyomorpha halys* naturally laid eggs were parasitized by *An. bifascatus*, *Tr. mitsukurii*, *Tr. basalis* and *Telenomus* sp. In addition, *An. bifasciatus*, *Tr. mitsukurii* and *Oo. telenomicida* successfully emerged from sentinel eggs exposed in the field. All of the five parasitoid species found during the survey are new records for Slovenia. *Anastatus bifasciatus* was the predominant and most abundant native species parasitizing *H. halys* eggs in the field, as already noted in previous European studies [36,37,38]. Egg parasitoids of the genus *Anastatus* were reported as the dominant native parasitoids in the United States [34,61] and in the native range of *H. halys* in China [32], where they are a part of the natural parasitoid community.

In 2020 the non-native parasitoid *Tr. mitsukurii* was found for the first time in Europe, and the discovery of this species in Western Slovenia is likely related to the range expansion of the species from the neighboring Italian region of Friuli Venezia Giulia [43,44]. Apparently, *Tr. mitsukurii* has followed the same pattern of introduction and spread in the region as its host [22]. Regularly invaders leave behind 75% or more of the parasite and pathogen species from their native range [62]. Nevertheless, it seems that the *H. halys* introduction provided some invasion opportunity for parasitoids from its native range. This phenomenon is particularly evident in species of *Trissolcus*, which have shown the growing trend in tracking their invasive host in recent years [43].

The dispersal of parasitoids from their native range can be accelerated by repeated introductions of the host species [63]. Although *Tr. japonicus* adventive populations during this study have not been detected in the region, it can be expected that this species will also expand into Slovenia in the near future. Bioclimatic envelope models predict that *Tr. japonicus* will follow its host throughout Europe, spreading within regions most suitable for its settlement located in southwestern France, northeastern Spain, northern Italy, western Slovenia and Croatia [64].

In addition to parasitoid detection, the main objective of the current study was the evaluation of their impact on the pest population under field conditions. The mean parasitism rates of naturally laid eggs during the two years were 3.0% and 14.4%, respectively. In 2020 it ranged from 7.1 to 30.1%, with high variability observed between locations, landscapes, plant host species and parasitoid species. The overall parasitism rate in the first year was based entirely on recruitment of native parasitoids, while in the second year, the non-native *Tr. mitsukurii* made an important contribution. An increasing trend in parasitism rate and rapid recruitment of native parasitoids onto the novel invading host was observed during the research, with *An. bifasciatus* as the most abundant and widespread species. It should be pointed out that *H. halys* was not present in the region until 2017, and by 2019, slow population growth was recorded. Therefore, native parasitoids’ adaptation to the new host may be occurring quickly. In contrast to our findings, Dieckhoff et al. [34], who performed long-term surveys in northern Delaware, in the Mid-Atlantic region of the United States, had no indication that parasitism by native parasitoids increased over the course of nine years. Furthermore, non-native *Trissolcus* species, which are known to be effective biocontrol agents of *H. halys* in its native range, indigenous species can play an important role in pest population reduction in an invaded area. This study supports previous work that suggested *An. bifasciatus* as a potential biological control agent of *H. halys* in Europe [33,40,41,65].

The main approach in augmentative biocontrol of *H. halys* with *An. bifasciatus* is to release a relatively small number of adults in orchards or row crops to accelerate natural populations of parasitoids at releasing sites and to control the pest in the short term. Augmentative biocontrol of *H. halys* with *An. bifasciatus* was tested over three years in four orchards in Switzerland and Italy. Considering the parasitism level only, with an average of 6%, this was not high enough to effectively suppress the pest. However, the overall impact of *A. bifasciatus* on the mortality of *H. halys* found in the study was actually greater. Together with pre-imaginal parasitoid mortality (3.3%) and host feeding (6%), the overall *H. halys* mortality could reach an average of 15% [41].

Adaptation to local environmental conditions and ecosystems is an important advantage of native biological control agents [66] when compared to classical biological control agents, which are known to be rapid in the control of the co-evolved exotic prey. The coexistence and synergistic action of native and non-native species in controlling *H. halys* seem to be feasible. Konopka et al. [67] already confirmed the coexistence of *Tr. japonicus* and *An. bifasciatus* under laboratory conditions. The coexistence of species could be the result of counterbalance competition. *Trissolcus japonicus* dominates as a superior extrinsic (adult stage) competitor, characterized by superior egg guarding and aggressiveness, while *A. bifasciatus* is a superior intrinsic (larval stage) competitor with the ability to develop in previously parasitized eggs of all stages. By parasitization and host feeding, *An. bifasciatus* can interfere with the development of *Tr. japonicus*. The recent accidental arrivals of two non-native parasitoids in Europe represents a great challenge and opportunity for the future biological control of *H. halys*, which also brings different risk factors to native species that should be assessed.

The complex of natural enemies attacking *H. halys* diffesr in diverse habitats. In developing successful control strategies for managing invasive exotic pests, the efficacy of natural enemy complexes needs to be evaluated in a variety of landscapes [68]. The comparison of parasitoid species composition between the different habitats investigated in our study demonstrated a predominance of the native generalist parasitoid *An. bifasciatus* in urban and suburban areas, while *Tr. mitsukurii* was the prevailing species in agricultural areas. Our results support those obtained by Sabbatini Peverieri et al. [43], Zapponi et al. [38] and Scaccini et al. [44] in Northeast Italy.

Urban vegetation seems to be very attractive for *H. halys*. Deciduous ornamental trees act as a shelter, food source and oviposition sites. Among the 21 plant hosts registered during the survey, in urban areas, *Catalpa bignonioides* and *Acer platanoides* stand out regarding the highest number of egg masses collected and the parasitism rates recorded. A season-long presence of all life stages of *H. halys* confirms its preference for those two hosts, which was also presented in other recent studies [2,27,37,69,70,71].

In our study, *Malus domestica* has been found as the main oviposition host in agricultural areas. Apple is reported as a common host for *H. halys* in its native range [72] and is among the most acceptable hosts in the invaded areas [26,72]. It is considered the early season host [1,70]. Compared to other fruit trees, it is a less optimal host but sufficient when an alternative host is not available [73]. The parasitoid community composed of *An. bifasciatus* and *Tr. mitsukurii* in equal proportions was identified in apple orchards in Western Slovenia, with an overall parasitism rate of 8.6%. The coexistence of two native parasitoids from different genera in a similar habitat has been previously noted by Herlihy et al. [74], who found *Anastatus reduvii* (Howard)and *Trissolcus euschisti* (Ashmead) emerging from *H. halys* sentinel eggs exposed in apple orchards. That study also confirmed that the parasitoids were habitat-specific.

In general, the lower parasitism rates found in agricultural areas are related to pest management techniques undertaken in orchards, including broad-spectrum insecticide treatments. The compatibility of biological control with insecticide application is a key concern for a natural enemy establishment and the impact on pests in agroecosystems [75]. Insecticide treatment has a great impact on the reduction of parasitoid emergence from parasite egg masses [76].

Sentinel egg mass exposure can be an important tool in discovering and confirming the existence of native and alien stink bug parasitoids. However, our findings agree with Jones et al. [61] that this method underestimates the actual rates of parasitism and does not provide the whole picture of the parasitoid community composition. The total number of parasitoids emerged from naturally laid *H. halys* egg masses, and the species richness was greater than that obtained from sentinel egg masses.

The overall impact of chewing and sucking predators on naturally laid eggs observed during this survey was around 6%, which is consistent with findings in northern Italy [38] and in the Mid-Atlantic region of the USA [35]. Interestingly, the percentage of predation found on sentinel egg masses was over two times higher than on naturally laid eggs. In similar studies, highly variable results were obtained. Some authors reported a significantly lower percentage of predation compared to our results [36,37], while in other studies, the impact of predation was higher [33,34,47,77,78]. The findings of various studies suggest that the predation rate is very likely influenced by habitat diversity and that overall predation rates increase with plant species richness.

Considering the identity of egg predators, hypotheses can be made on the basis of laboratory studies performed in Italy [79,80], indicating that freshly laid *H. halys* egg masses are mainly preyed upon by some orthopterans, coccinellids and reduviids such as *Eupholidoptera chabrieri* (Charpentier), *Adalia bipunctata* (Linnaeus) and *Nagusta goedelii* (Kolenati), respectively [79], whereas ants such as *Crematogaster scutellaris* (Olivier) do not consume eggs, despite being able to prey upon all juvenile instars [80]. In any case, egg predators make an important contribution to the overall reduction of the *H. halys* population in natural habitats. Therefore, their impact should not be underestimated, and specific field studies are necessary to better clarify their role in the context of conservation biocontrol.

The identification of native natural enemies associated with *H. halys* and evaluation of their impact in its invaded range is essential prior to biological control implementation. The present study provides detailed information on the presence and species diversity of egg parasitoids associated with *H. halys* in Western Slovenia and the first insight into their performance in reducing *H. halys* populations. The data obtained provide a solid basis for future activities regarding the biological control of *H. halys* in Slovenia.

## Figures and Tables

**Table 1:** Data on field survey in 2019 and 2020 in the Goriška region (Western Slovenia).

No.	Location Name	GPS Coordinates	Type of Location	Predominant Host Plants
1	Kromberk	45°57′46.7964″ N 13°39′29.7324″ E	Suburban area (backyard orchards and vineyards, hedges and shrubs)	*Vitis vinifera*, *Prunus avium*, *Olea europea*, *Prunus domestica, Actinidia deliciosa, Cornus sanguinea*, *Ligustrum vulgare, Acer negundo*, *Hydrangea macrophylla*
2	Šempeter pri Gorici	45°55′55.3908″ N 13°38′42.6408″ E	Suburban area/ Agricultural area (intensive fruit orchards, ornamental plants)	*Catalpa bignonioides, Parthenocissus tricuspidata*, *Actinidia deliciosa*, *Malus domestica*, *Pyrus pyrifolia*, *Pyrus communis*, *Olea europea*, *Prunus dulcis*, *Corylus avellana*, *Prunus persica*, *Vitis vinifera*, *Hibiscus syriaticus*, *Buddleja davidii*
3	Miren	45°53′20.5116″ N 13°35′37.7916″ E	Agricultural area (intensive fruit orchards, hedges and shrubs)	*Malus domestica*, *Pyrus communis*, *Corylus avellana*, *Cornus sanguinea*, *Ligustrum vulgare*, *Acer negundo*,*Clematis vitalba*, *Ailanthus altissima*, *Parthenocissus quinquefolia*
4	Dombrava	45°53′41.406″ N 13°40′54.3144″ E	Agricultural area (intensive fruit orchards)	*Malus domestica, Pyrus communis, Prunus persica, Vitis vinifera, Zea mays, Cornus sanguinea*
5	Goriška Brda	46°0′11.6532″ N 13°31′23.9376″ E	Suburban area/ Agricultural area	*Solanum lycopersicum*, *Vitis vinifera*, *Prunus avium*, *Monarda didyma*
	Goriška Brda I.	45°58′31.9584″ N 13°31′53.3352″ E	Agricultural area (intensive fruit orchards)	*Olea europea*, *Prunus avium, Vitis vinifera*
6	Stara Gora	45°55′48.5724″ N 13°40′50.0052″ E	Agricultural area (extensive fruit orchard)	*Actinidia deliciosa*, *Vitis vinifera*, *Robinia pseudoacacia*, *Acer* sp., *Paulownia tomentosa*
7	Pristava	45°56′48.2928″ N 13°38′41.2476″ E	Suburban area (extensive fruit orchard)	*Actinidia deliciosa*, *Corylus avellana*, *Paulownia tomentosa*
8	Potoče	45°52′33.3732″ N 13°49′20.3016″ E	Agricultural area (field crops, vineyards)	*Zea mays*, *Vitis vinifera*
9	Bilje	45°53′47.3856″ N 13°37′27.2604″ E	Urban area (ornamental trees)	*Acer plantanoides*
10	Nova Gorica	45°57′17.3016″ N 13°39′12.1788″ E	Urban area (ornamental trees)	*Catalpa bignonioides*, *Acer plantanoides*
11	Dobravlje	45°52′16.6152″ N 13°50′9.816″ E	Agricultural area (field crops)	*Zea mays*

**Table 2:** Data on *Halyomorpha halys* naturally laid eggs collected in 2019 and 2020 in different locations in the Goriška region (Western Slovenia), percentage of hatched/unhatched/predated/parasitized eggs and number of parasitoids emerged.

No.	Location Name	Year	No. of Egg Masses	No. of Eggs	% Hatched	% Unhatched	% Predated	% Parasitized	No. of Parasitoids Emerged
1	Kromberk	2019	3	84	66.67	14.29	0,0	19.05	16 AB
	Kromberk	2020	7	196	71.94	6.63	2.04	19.39	32 AB
2	Šempeter	2019	8	219	89.50	4.10	6.39	0.0	0
	Šempeter	2020	69	1759	66.85	11.31	7.39	14.38	104 AB, 71 TM, 4 Tel
3	Miren	2019	4	109	81.65	3.67	14.68	0.0	0
	Miren	2020	22	549	67.76	13.84	2.03	16.39	9 AB, 4 TM
4	Dombrava	2019	2	57	87.72	7.02	5.26	0.0	0
	Dombrava	2020	19	502	73.11	19.32	7.57	0.0	0
5	Goriška Brda	2019	2	57	92.98	7.02	0.0	0.0	0
	Goriška Brda	2020	7	196	77.04	4.08	0.0	18.88	24 AB, 12 TB
6	Stara Gora	2020	4	111	75.68	9.91	0.0	14.41	2 AB
7	Pristava	2020	8	195	69.74	7.18	9.23	13.85	6 AB, 10 Tel
8	Potoče	2020	3	83	65.06	8.43	0.0	26.50	18 AB
9	Bilje	2020	10	266	53.01	6.77	10.15	30.06	33 AB
10	Nova Gorica	2020	5	137	76.64	9.49	0.0	13.87	17 AB
11	Dobravlje	2020	1	28	35.71	57.14	0.0	7.14	2 TM
**Total**		2019	19	526	84.41	6.2	6.2	3.04	16
		2020	155	4075	68.31	11.66	5.60	14.43	316

AB = *Anastatus bifasciatus*, Tel = *Telenomus* sp., TM = *Trissolcus mitsukurii*, TB = *Trissolcus basalis*.

**Table 3:** Data on *H. halys* sentinel eggs exposed in 2019 and 2020 in two locations in the Goriška region (Western Slovenia), percentage of hatched/unhatched/predated/parasitized eggs and on data on parasitoids emerged.

Year	Location	Location Type	No. of Egg Masses	No. of Eggs	% Hatched	% Unhatched	% Predated	% Parasitized	No. of Parasitoids Emerged
2019	Kromberk	Suburban area	20	530	71.69	17.74	10.57	0	0
	Miren	Agricultural area	20	528	69.32	12.88	17.42	0.38	2 OT
**Total**			40	1058	70.51	15.31	13.99	0.19	
2020	Kromberk	Suburban area	50	1340	60.67	20.97	11.49	6.86	22 AB, 70 TM
	Miren	Agricultural area	41	1077	60.63	16.99	20.98	1.39	15 AB
**Total**			91	2417	60.65	19.20	15.71	4.42	

OT = *Ooencyrtus telenomicida*, AB = *Anastatus bifasciatus*, TM = *Trissolcus mitsukurii*.

## Supplementary Materials

The following are available online at https://www.mdpi.com/article/10.3390/insects12060505/s1, Table S1: Data on *H. halys* oviposition hosts in agricultural, suburban and urban area found in Goriška region (Western Slovenia).
