# Supplementary material for: Native and Non-Native Egg Parasitoids Associated with Brown Marmorated Stink Bug (Halyomorpha halys [Stål, 1855]; Hemiptera: Pentatomidae) in Western Slovenia
Source: Insects. 2021 May 31;12(6):505. doi: 10.3390/insects12060505 (PMC8228850; doi:10.3390/insects12060505)
Supplement: Supplementary file 1 [file insects-12-00505-s001.zip › insects-1186221-supplementary.pdf]

Table S1. Data on *Halyomorpha halys* oviposition hosts in agricultural, suburban and urban area found in Goriška region (Western Slovenia).

Area	Host plant	No. of Egg Masses Collected	No. of Eggs	No. of Egg Masses Parazitzated	No. of Eggs Parazitizated	Parasitism Rate (%)	Parasitoid Species Composition (%)			
							AB	TM	TB	Tel
Agricultural area	*Malus domestica*	41	1052	5	90	8.6	51.1	48.9	0	0
	Actinidia deliciosa	17	460	4	30	6.5	100.0	0	0	0
	Olea europea	24	600	9	92	15.3	88.0	16.0	0	0
	Pyrus pyrifolia	7	197	3	30	15.2	0	100.0	0	0
	Corylus avellana	8	215	1	1	0.5	100.0	0	0	0
	Prunus avium	4	112	0	0	0.0	0	0	0	0
	Zea mays	4	111	2	24	21.6	91.7	8.3	0	0
	Vitis vinifera	1	28	0	0	0.0	0	0	0	0
	Pyrus communis	2	56	0	0	0.0	0	0	0	0
	Prunus dulcis	1	28	0	0	0.0	0	0	0	0
	Clematis vitalba	1	28	0	0	0.0	0	0	0	0
	Robinia pseudoacacia	1	28	0	0	0.0	0	0	0	0
**TOTAL**		**111**	**2747**	**24**	**267**	**9.7**				
Suburban area	*Actinidia deliciosa*	6	155	2	22	14.2	81.8	0	0	18.2
	Olea europea	1	27	1	22	81.5	100.0	0	0	0
	Corylus avellana	1	14	1	13	92.9	0	00	0	100.0
	Vitis vinifera	2	42	2	30	71.4	53.3	46.7	0	0
	Prunus domestica	2	56	1	28	50.0	100.0	0	0	0
	Solanum lycopersicum	1	26	1	13	50.0	0	0	100.0	0
	Catalpa bignonioides	18	494	5	99	20.0	14.1	85.9	0	0
	Paulownia tomentosa	2	54	1	12	22.2	100.0	0	0	0
	Monarda didyma	1	26	1	2	7.7	100.0	0	0	0
	Parthenocissus tricuspidata	9	249	0	0	0.0	0	0	0	0
	Buddleja davidii	1	28	0	0	0.0	0	0	0	0
	Hydrangea macrophylla	1	28	0	0	0.0	0	0	0	0

	Hibiscus syriacus	1	27	0	0	0.0	0	0	0	0
**TOTAL**		**46**	**1226**	**15**	**241**	**19.7**				
**Urban area**	*Catalpa bignonioides*	5	137	1	19	13.9	100.0	0	0	0
	Acer platanoides	12	321	*5*	80	24.9	100.0	0	00	0
TOTAL		17	458	6	99	21.6				

AB = *Anastatus bifasciatus*, TM = *Trissolcus mitsukurii*, TB = *Trissolcus basalis*, Tel = *Telenomus* sp.
